# The Impact of Disease-Modifying Therapy Access Barriers on People With Multiple Sclerosis: Mixed-Methods Study

**DOI:** 10.2196/11168

**Published:** 2018-10-30

**Authors:** Kristina F Simacek, John J Ko, Debbie Moreton, Stefan Varga, Kristen Johnson, Bozena J Katic

**Affiliations:** 1 Research and Development PatientsLikeMe Cambridge, MA United States; 2 Health Economic and Outcomes Research Novartis Pharmaceuticals Corporation East Hanover, NJ United States; 3 PatientsLikeMe Cambridge, MA United States; 4 Jefferson College of Population Health Thomas Jefferson University Philadelphia, PA United States

**Keywords:** cost sharing, insurance, mixed methods, multiple sclerosis, out-of-pocket costs, patient adherence, pharmaceutical services, self-report, surveys and questionnaires

## Abstract

**Background:**

In the United States, people with relapsing-remitting multiple sclerosis (RRMS) can face difficulty accessing disease-modifying therapies (DMTs) because of insurance, pharmacy, or provider policies. These barriers have been associated with poor adherence and negative health outcomes.

**Objective:**

The goals of this study were to describe the overall occurrence of difficulties and delays associated with gaining access to DMTs among people with RRMS, to assess DMT adherence during periods of reduced access, and to contextualize the patients’ journey from receipt of a prescription for DMT to obtaining and taking their medication when faced with access barriers.

**Methods:**

We recruited US-based adults self-reporting RRMS from a Web-based health data-sharing social network, PatientsLikeMe. Individuals were invited to complete a Web-based survey if they reported a diagnosis of RRMS and were prescribed a DMT for MS. Follow-up phone interviews were conducted with 10 respondents who reported experiencing an MS-related relapse during the time they had experienced challenges accessing DMTs.

**Results:**

Among 507 survey completers, nearly half were either currently experiencing an issue related to DMT assess or had difficulty accessing a DMT in the past (233/507, 46.0%). The most frequently reported reasons for access difficulty were authorization requirements by insurance companies (past issues: 78/182, 42.9%; current issues: 9/42, 21%) and high out-of-pocket costs (past issues: 54/182, 29.7%; current issues: 13/42, 31%). About half (20/39, 51%) of participants with current access issues and over a third (68/165, 41.2%) of those with past issues went without their medication until they could access their prescribed DMT. Relapses were reported during periods of reduced DMT access for almost half (56/118, 47.5%) of those with past issues and nearly half (22/45, 49%) of those with current issues. Resolving access issues involved multiple stakeholder agents often coordinated in a patient-led effort. Among those who had resolved issues, about half (57/119, 47.9%) reported that doctors or office staff were involved, under half (48/119, 40.3%) were involved themselves, and about a third (39/119, 32.8%) reported the drug manufacturer was involved in resolving the issue. Follow-up interviews revealed that the financial burden associated with obtaining a prescribed DMT led to nonadherence. Additionally, participants felt that DMT treatment delays and stress associated with obtaining the DMT triggered relapses or worsened their MS.

**Conclusions:**

This study expands current research by using a patient-centered, mixed-methods approach to describe barriers to MS treatment, the process to resolve barriers, and the perceived impact of treatment barriers on outcomes. Issues related to DMT access occur frequently, with individuals often serving as their own agents when navigating access difficulties to obtain their medication(s). Support for resolution of DMT access is needed to prevent undue stress and nonadherence.

## Introduction

Multiple sclerosis (MS) is a chronic autoimmune disease of the central nervous system affecting an estimated 450,000-550,000 people in the United States [1,2]. The financial burden accompanying MS is a central component of the disease experience, ranking second among all chronic conditions in direct costs behind congestive heart failure [3]. In addition to the impact of decreased productivity and unemployment [4-6], patients often shoulder the burden of high out-of-pocket costs for medications, tests, magnetic resonance imaging (MRI), medical equipment, and inpatient or outpatient visits [3,7,8].

Disease-modifying therapies (DMTs) slow the disease progression and related disability and are the prevailing treatment for people with MS [9]. Responding to rising costs of DMTs [10,11], insurers have adopted strategies to optimize the utilization of specialty medications through management, including requiring prior authorization, step therapy (where a patient must fail a preferred medication first), or tiered formularies that increase cost sharing for patients [12,13]. Falling into the “specialty drug” tier of most benefits plans, DMTs are subject to higher out-of-pocket costs (ie, tiers that require high coinsurance or copayment) than nonspecialty drugs [14]. Due to the clinical benefits of specialty drugs like DMTs, their use remains relatively insensitive to such cost-sharing programs [14,15]. For people with MS, high out-of-pocket costs for DMTs have been associated with lower adherence and inappropriate disease management, which leaves patients at increased risk of relapse and payers vulnerable to increased associated resource use [16,17].

A growing body of research has explored the impact of current insurance coverage and related barriers to DMT access on people with MS. An estimated 25% of people with MS taking DMTs receive these drugs at little to no cost through pharmaceutical company financial aid programs, and such programs have been shown to increase the adherence [18,19]. Discounted drug programs can be essential in mitigating prohibitive financial barriers for patients; however, regulations may prevent those with government-based insurance coverage from using these programs [20], so they may not be an ideal solution for all patients.

One understudied aspect of access to MS DMTs is the patient experience in navigating the treatment acquisition landscape. Although prior research has focused on the number of people receiving DMTs, how they paid for their treatment, and insurance-related access barriers [19], it is not yet clear how people with MS perceive and navigate the current health care system to obtain their medication and how they perceive this impacts their health outcomes and self-reported quality of life.

The goals of this study are to describe the overall occurrence of difficulties and delays associated with gaining access to DMTs among people with MS, to assess the impact of access barriers on DMT medication adherence and MS outcomes, and to contextualize the experience of obtaining a DMT after the receipt of a prescription.

## Methods

### Study Design

This mixed-methods study included a population of people with MS who were members of PatientsLikeMe as of February 2016. PatientsLikeMe is a real-time, data-sharing, and research platform of patient communities devoted to a variety of life-changing health conditions. As of July 1, 2018, PatientsLikeMe had >600,000 members with >2800 different health conditions, including 59,853 members with MS, and among those reporting their variant of MS, most (32,732/50,868, 64.35%) reported having relapsing-remitting MS (RRMS). Of note, participants were not compensated. This study was exempted from review by the New England Independent Review Board on February 26, 2016 (study #16-082).

The study was designed into 2 distinct phases, consisting of a Web-based survey and qualitative interviews following the survey completion with a subset of survey participants who had experienced a relapse during their access issue.

### Phase 1: Web-Based Survey

A sample of people with RRMS from the PatientsLikeMe community participated in a cross-sectional, Web-based questionnaire fielded by PatientsLikeMe [21]. A closed design was used, meaning only members of PatientsLikeMe who were invited to take part and who had logged in with their password could see the survey. An informed consent document was displayed prior to starting the survey, and a waiver of documentation of informed consent was obtained from the New England Independent Review Board. The survey was administered electronically for 4 weeks in February and March 2016.

#### Survey Development

The survey included demographics and a series of questions concerning experiences with access to DMTs. We used 3 mutually exclusive branching options to segment respondents who (1) were currently experiencing a DMT access barrier, (2) had past (but not current) barrier accessing DMTs, and (3) never experienced an access barrier to their DMTs. Preliminary survey items were derived from targeted literature review and consultation with MS experts; a PatientsLikeMe patient member (DM) provided feedback on items, survey length, and participant interpretability. Prior to fielding the survey, it went through a series of reviews among investigators and was electronically pretested for design elements, question ordering, and flow; see Multimedia Appendix 1 for survey questions.

#### Survey Population

Eligible participants were aged ≥18 years, who self-reported residence within the United States, reported a physician diagnosis of RRMS, and had a recent (90-day) activity on the PatientsLikeMe website. Eligible participants were sent a private survey invitation through the PatientsLikeMe website. Notably, participants were screened out if they did not confirm the above eligibility criteria or reported never being prescribed a DMT for RRMS.

#### Measures

*DMT access barriers* included any of the following: lack of insurance; insurance not covering the DMT; prior authorization documentation requirement; high out-of-pocket costs; requirement to take another DMT before the prescribed DMT (ie, step through); inability to obtain the DMT at their desired pharmacy or infusion center; or other difficulties.

*Adherence* was measured by asking participants how often they take their medication as prescribed. Responses could be given in 10% increments from 0% (none of the time) to 100% (always).

*Health literacy* was measured using respondents’ level of confidence filling out medical forms by themselves [22,23]. In addition, a dichotomous variable was created to classify those who were “quite a bit” or “extremely” confident as having high health literacy; those who responded “somewhat,” “a little,” or “not at all” as having low health literacy.

*Stress* was measured on a 10-point numeric scale. Respondents who had experienced an access barrier were asked how stressful their most recent DMT access issue was from 0 (not at all) to 10 (greatly). Those who had not experienced an access issue were instead asked about their stress level in the last 7 days.

In addition to survey responses, most recent Patient-Determined Disease Steps (PDDS) score [24-27] was obtained from participants’ PatientsLikeMe profiles. PDDS is measured on a 9-point scale from 0 (Normal) to 8 (Bedridden).

#### Analyses

In this study, subgroups (current, past, or never had DMT issue) were defined by the following 2 questions: “Which option best describes your experience with accessing or receiving your DMT medication for MS?” (current DMT access difficulty, a past difficulty, or never had a difficulty) and “Have you ever had any of the following difficulties accessing or receiving DMT medications for MS?” (a select-all-that-apply list including items such as covered by insurance plan and it required authorizing documentation).

During analysis, it was discovered that some participants reported they had never experienced a DMT access issue; however, when queried about specific access issues, they selected a specific DMT access barrier (eg, authorizing documentation, high out-of-pocket costs, medications not covered by insurance). For clarification, a 2-question follow-up survey was fielded in June and July 2016 to 108 respondents who fell into this category.

The first question was closed-ended: “Were any of the following situations burdensome enough to delay or prevent you from getting your DMT medication? (check all that apply).” Answer options included (1) having to fill out paperwork or get other documentation for DMT access or (2) DMT out-of-pocket costs too high. The second question was open-ended: “Please tell us a little bit more about this difficulty and the effect it had on you (if any).”

Variables with closed-ended response options were analyzed using descriptive and summary statistics. We used Wilcoxon rank-sum tests to compare groups with nonnormally distributed values; *t* tests were used to compare groups with normally distributed values. An alpha value of ≤.05 was considered significant. Statistical analyses were performed using SAS software, version 9.4 (Cary, NC, USA). Open-ended questions were coded for themes using conventional content analysis in ATLAS.ti software (ATLAS.ti Scientific Software Development GmbH, Berlin, Germany, version 5.0) [28,29].

#### Data Exclusion

A validated sample of survey respondents excluded 121 respondents from the analyzed sample who gave conflicting responses to the survey branches. The total number of respondents in the “never” category reduced from the full sample of 507 respondents to 386.

### Phase 2: Qualitative Interviews

Interview methods are described using the consolidated criteria for reporting qualitative research [30]. During the second phase of this study, 10 respondents who completed the questionnaire were selected to participate in a single, 60-minute follow-up interview between April and June 2016. Interviews were conducted at the participants’ convenience via phone or videoconference by researchers (KFS and BJK). The interviewers had no prior relationship with interviewees. Interviews were audiorecorded and transcribed. Participants were not given transcripts or findings for review.

#### Interview Sample Population

Participants were selected for interviews based on their survey response, reporting either a current or past issue with DMT access, at least one MS relapse during the period of their DMT access difficulty, and consent to a follow-up interview in the first survey.

#### Interview Guide Development

The goal of the interview was to supplement information collected in the questionnaire, among participants who had experienced DMT access barriers and a negative health event (eg, MS relapse) during the assess issue, using qualitative data (see Multimedia Appendix 2 for the Interview Guide). The following 4 research questions directed the interview guide: (1) How did people resolve their DMT access issues? (2) What are the clinical consequences of DMT access barriers? (3) What are the impacts of DMT access issues on quality of life? and (4) Who are the agents involved in helping people access their DMT?

#### Analyses

Interviews were analyzed for themes by researchers using a constant comparative method [31,32]. To represent interactions between study participants and the agents or organizations involved in resolving their access to DMTs, an aggregate egocentric social network map was created based on the interviews [33,34]. To create the map, an iterative process was used to draw connections between each interviewee and others involved in DMT access resolution. Each subsequent interviewee was compared with the previous ones until all connections mentioned in the interviews were placed on the generalized map, with the “ego” (study participant) in the center.

## Results

### Survey Results

#### Survey Sample

Of 5239 people invited to the survey, 947 viewed the invitation and 584 participated in the survey, for a participation rate of 61.6%. A total of 507 participants completed the survey, for a completion rate of 86.8% (507/584). A subset of 40 participants (out of 108 who were invited) completed the follow-up survey, for a participation rate of 85%). Among survey completers, 78.3% (397/507) were females, 89.6% (441/507) were white, and participants were, on average, 49 years old (Table 1). Of the survey completers, 66% (234/507) had high health literacy; however, the percentage of those with high health literacy was lowest among those currently experiencing access issues (25/45, 56%, of those with a current issue vs 138/188, 73.4%, of those with a past issue and 127/153, 83.0%, of those who never had issues had high health literacy). This difference was statistically significant (*χ*^2^_1_=14.6, *P*<.001). Median PDDS score was 4 (range, 1-8; interquartile range [IQR], 3.0), indicating some gait disability. Of those in the validated sample, 60.4% (233/386) had experienced difficulty accessing a DMT in the past (188/386, 48.7%) or were currently experiencing an issue related to DMTs access (45/386, 11.7%). Average time to receive the originally prescribed DMT after experiencing an access barrier was 8.3 (SD 16.5) weeks.

#### Reasons for Disease-Modifying Therapy Access Difficulties

The most frequently reported reasons for DMT-related access difficulties were “insurance required authorizing documentation” (9/42, 21.4%, current issue and 78/182, 42.9%, past issue) and “high out-of-pocket costs” (13/42, 31.0%, current issue and 54/182, 29.7%, past issue; Table 2). Other reasons included administrative coordination problems among insurance companies, pharmacies, and clinician’s offices.

Among the 40 follow-up respondents, 60% (24/40) clarified that the DMT access barrier delayed or prevented them from getting their DMT medication. The reasons for access problems mirrored the results obtained from the full survey, with about half of those who had experienced barriers attributing them to “authorizing documentation” (58%, 14/24), a third to “high out-of-pocket costs” (33%, 8/24), and several to “not covered by insurance” (8%, 2/24). Furthermore, a slight majority of the 40 follow-up respondents (66%, 16/24) confirmed that their access difficulty did not delay or prevent them from getting their DMT.

#### Adherence to Prescribed Disease-Modifying Therapies During Periods of Decreased Access

Respondents frequently went without any RRMS medication until they could obtain their prescribed DMT. Nearly half (68/165, 41.2%) of respondents who had experienced a past access issue reported going without their medication(s) until they could access their prescribed DMT, 4.8% (8/165) switched to a different DMT, 1.8% (3/165) continued their old medication, and 1.2% (2/165) received a different DMT until receiving the originally prescribed DMT. Among respondents currently experiencing a DMT access issue, about half (20/39, 51%) responded they were not currently taking any medications for their MS, 23% (13/39) continued their old medication, 13% (5/39) took a newly prescribed DMT, and 3% (1/39) reported they were instead taking a newly prescribed non-DMT medication for MS (Table 3).

Self-reported adherence to DMT medication during an access barrier (mean 8.97 [SD 2.47]) was significantly lower than self-reported typical DMT adherence (mean 9.61 [SD 1.0]). A paired *t* test showed that this difference was statistically significant (*t*_101_=−2.48, *P*=.02).

#### Outcomes During Periods of Decreased Disease-Modifying Therapy Access: Stress and Multiple Sclerosis Relapse

Among respondents who experienced a DMT access barrier, 49% (22/45) of those with a current access issue reported at least one MS relapse during the time of the barrier; 29.8% (56/188) self-reported at least one MS relapse during a past DMT access issue. The Wilcoxon rank-sum tests showed significantly higher stress levels among those who experienced at least one MS relapse during a past access issue (n=48; median, 8.5; IQR, 7.0-10.0) than among those who did not experience relapse (n=113; median, 7.0; IQR, 5-9; *Z*=3.228, *P*=.001); this effect did not reach significance for those with a current issue (n=19; median, 10.0; IQR, 7.0-10.0 vs n=20; median 7.0; IQR, 4.3-8.8; *Z*=−1.835, *P*=.08).

#### Stakeholder Agents Involved in Disease-Modifying Therapy Access

Among respondents who experienced past difficulties gaining access to DMTs, 47.9% (57/119) involved doctors or office staff to help resolve the DMT access issue and 40.3% (48/119) said they were at least partially responsible for resolving the issue themselves. The remaining agents involved in resolving the issue were the drug manufacturers (39/119, 32.8%), pharmacy or specialty pharmacy (31/119, 26.1%), insurance companies (26/119, 21.8%), and infusion centers (6/119, 5.0%). Few caregivers were involved in resolving the access barriers (2/119, 1.7%).

**Table 1:** Participant characteristics.

Characteristic			Total completed (N=507)		Validated sample^a^ (n=386)		Current issue (n=45)		Past issue (n=188)		Never had issue (n=153)
Age (years), mean (SD)			49.1 (10.4)		49.7 (10.2)		50.2 (9.7)		48.9 (10.8)		50.5 (9.7)
Female, n (%)			397 (78.3)		308 (79.8)		39 (86.7)		154 (81.9)		115 (75.2)
White, n (%)			441 (89.6)		335 (89.8)		40 (90.9)		161 (88.5)		134 (91.2)
**Ethnicity, n (%)**											
	Not Hispanic	462 (94.7)		357 (95.7)		41 (93.2)		173 (95.6)		143 (96.6)	
Number of comorbidities, median (range, IQR^b^)			1 (1-27, 1.0)		1 (1-27, 2.0)		1 (1-21, 3.0)		1 (1-27, 1.0)		1 (1-15, 1.0)
**Education^c^, n (%)**											
	High school or less	53 (12.7)		39 (12.2)		6 (16.2)		21 (13.6)		12 (9.4)	
	Some college	165 (39.6)		126 (39.4)		18 (48.6)		56 (36.1)		52 (40.6)	
	College degree	131 (31.4)		107 (33.4)		5 (13.5)		57 (36.8)		45 (35.2)	
	Postgraduate work	68 (16.3)		48 (15.0)		8 (21.6)		21 (13.6)		19 (14.8)	
**High Health Literacy^d^, n (%)**											
	Quite a bit or extremely	234 (66.1)		182 (65.5)		25 (55.5)		138 (73.4)		127 (83.0)	
**Health Insurance Status^e^, n (%)**											
	Employer based	209 (47.5)		162 (47)		18 (43.9)		76 (45.5)		68 (49.6)	
	Direct	34 (7.7)		26 (7.5)		2 (4.9)		14 (8.4)		10 (7.3)	
	Medicare	131 (29.8)		103 (29.9)		10 (24.4)		56 (33.5)		37 (27.0)	
	Medicaid	38 (8.6)		32 (9.3)		8 (19.5)		11 (6.6)		13 (9.5)	
	Military	7 (1.6)		7 (2.0)		1 (2.4)		3 (1.8)		3 (2.2)	
	Veterans Affairs	9 (2.1)		8 (2.3)		1 (2.4)		2 (1.2)		5 (3.6)	
	None	8 (1.8)		4 (1.2)		1 (2.4)		3 (1.8)		0 (0)	
	Other	4 (0.8)		3 (0.8)		0 (0)		2 (1.2)		1 (0.7)	
PDDS^f^ score, median (range, IQR)			4 (1-8, 3.0)		4 (1-8, 3.0)		4 (1-8, 3.0)		4 (1-8, 3.0)		4 (1-8, 4.0)
Had relapse during access issue (self-reported), n (%)			N/A^g^		N/A		22 (48.9)		56 (29.8)		N/A
Average delay in weeks^h,i^, mean (SD)			N/A		N/A		N/A		8 (16.5)		N/A

^a^Subgroups (current, past, or never had a disease-modifying therapy [DMT] issue) were drawn from a validated sample based on the questions: “Which option best describes your experience with accessing or receiving your DMT medication for MS?” “Never” includes only those who selected none of the DMT access issues and “never” to “Which option best describes your experience with accessing or receiving your DMT medication for MS?”

^b^IQR: interquartile range.

^c^Validated sample, n=320

^d^Validated sample, n=278

^e^Validated sample, n=345

^f^PDDS: Patient Determined Disease Steps.

^g^N/A: not applicable.

^h^Asked only of those who eventually received the originally prescribed DMT.

^i^Past issue, n=64.

**Table 2:** Reported reasons for the disease-modifying therapy (DMT) access issue.

Source of access issue^a^	Past issue^b^, n (%)	Current issue^c^, n (%)	Follow-up^d^, n (%)
Insurance required authorizing documentation	78 (42.9)	9 (21.4)	14 (58.3)
High out-of-pocket costs	54 (29.7)	13 (31.0)	8 (33.3)
Not covered by my insurance plan	20 (11.0)	8 (19.0)	2 (8.3)
I do not have insurance	17 (9.3)	3 (7.1)	N/A^e^
Not at my desired pharmacy or infusion	14 (7.7)	2 (4.8)	N/A
Required to take one additional DMT	9 (4.9)	6 (14.3)	N/A
Other^f,g^	37 (20.3)	8 (19.0)	N/A
I don’t know	12 (6.6)	6 (14.3)	N/A

^a^Among a validated sample of those with past or current difficulties, access reasons were only asked of those who received a DMT prescription from their doctor. Question text for current issue was: “What difficulties are you having accessing or retrieving your DMT medication? Check all that apply”; question text for past access issue was: “Thinking about your most recent MS DMT access issue, what difficulties did you have accessing or receiving your DMT medication? Check all that apply.”

^b^Past issue, n=182.

^c^Current issue, n=42.

^d^Follow-up, n=24.

^e^N/A: not applicable.

^f^Other past reasons included administrative problems (n=9), provider changed or could not authorize (n=5), insurance or pharmacy denied drug or changed policies (n=5), insurance status change (n=4), appointment or prescription delay by the provider (n=4), paperwork issue (n=2), and other (n=5).

^g^Other current reasons included insurance policy changes or coverage loss (n=2), doctor or hospital problems (n=2), administrative problems (n=1), and switched drug (n=1).

**Table 3:** Medication status during past and current access issue.

Medication status^a^	Past issue^b^, n (%)	Current issue^c^, n (%)
Not taking any medication or went without medication	68 (41.2)	20 (51.3)
I received my disease-modifying therapy (DMT) medication within a reasonable amount of time,	56 (33.9)	N/A^d^
I was prescribed a new DMT instead	8 (4.8)	5 (12.8)
I continued taking my old medication	3 (1.8)	13 (33.4)
Received another DMT before receiving my prescribed DMT	2 (1.2)	N/A
I am taking a newly prescribed other non-DMT medication	N/A	1 (2.6)
Other	28 (17.0)	N/A

^a^Asked of respondents who had insurance or did not answer that difficulty obtaining DMT at a pharmacy or infusion center was their primary DMT access reason. Question text for current access status was: “What other MS medication(s) are you taking while your DMT medication access issue is being resolved?”; question text for past access issue was “Pick the option that best describes how your most recent MS DMT access issue was resolved.”

^b^Past issue, n=165.

^c^Current issue, n=39.

^d^N/A: not applicable.

### Qualitative Interviews

#### Interview Sample

Qualitative interviews were conducted among 10 survey respondents who experienced at least one MS relapse during a past or current period of decreased access to DMTs. Participants were predominantly females (9/10, 90%) with the mean age of 54 (range, 42-64) years; of them, 5 reported being on Medicaid or Medicare, 3 on employer-sponsored insurance, 1 did not specify insurance type, and 1 had no insurance. The median duration of MS was 9.5 (range, 2-15) years.

#### Themes From Qualitative Interviews

We identified several themes in the interviews; detailed examples are shown in Table 4.

##### Theme 1: Financial Burden Begins Prior to the Disease-Modifying Therapy Access Barrier and Can Impact Adherence to the Therapy

Many participants reported that prior to being prescribed a DMT, they had been in financial distress due to MS diagnostic costs and/or loss of income due to inability to work because of their symptoms (Table 4). For example, one participant said she had spent “all our savings” (Female, age 58 years) on medical costs related to her MS diagnosis, such as MRI tests. Another filed for bankruptcy because of bills stemming from his initial MS diagnosis. Most interviewees had stopped or reduced paid work because of MS symptoms, and over half (6/10, 60%) sought or received disability pay or subsisted on a fixed monthly income lower than the amount of their monthly copayment for their DMT medication. Most interviewees (8/10, 80%) went without DMT medication during their access barrier; the remaining 2 took a DMT they had been prescribed in the past until the access barrier was resolved.

##### Theme 2: Disease-Modifying Therapy Access Barriers are Associated With Stress and Relapses

Many participants felt delays in DMT treatment, and the stress associated with the process of obtaining the DMT triggered relapses or worsened their MS. As one participant who experienced a combination of billing errors and finding her DMT out of stock at her infusion center asked, “Why do *I* have to deal with this crap? You know how they say that stress makes it worse?” (Female, age 56 years). Several participants experienced worsening fatigue and cognitive problems related to MS and the lack of DMT, making efforts to resolve their access issue more difficult.

##### Theme 3: Disease-Modifying Therapy Access Issues Affect Quality of Life

Access difficulties impacted multiple facets of participants’ lives; emotional and interpersonal impacts were commonly mentioned. Emotional impacts included situational problems like “frayed nerves” (female, age 58 years) and exacerbation of pre-existing mental health comorbidities, such as depression. In addition to the time and effort spent on trying to access a DMT, several participants reported that the uncertainty of having unstable health because of going without a DMT made it difficult to schedule social events in advance.

##### Theme 4: Personal Resources Enable Access to Overcome Disease-Modifying Therapy Access Barriers

Several participants who succeeded in obtaining their DMT reported that they leveraged knowledge and skills from working in medical billing and other health care areas to expedite the process. This high level of health care literacy obtained through work experience facilitated their ability to document the issue and reach the appropriate agents who could help resolve the problem quickly.

**Table 4:** Themes arising from participant interviews.

Theme	Example quotes
Financial burden begins prior access issue and impacts the disease-modifying therapy (DMT) adherence	*I had to declare bankruptcy because of my first doctor’s bill. We accumulated US $15,000 in debt through the MRIs because they only cover half of one MRI per year and I had 6 that first year…I went from making US $6500 a month to US $1400 a month [on disability] with two kids and a vehicle. I got a little part time job that’s 12 hours a week, US $10 an hour. That pays for my drugs.* [Male, age 42 years] *Patients are not getting help. We cannot afford insurance. My discretionary income is US $10, that’s why I needed a physician who accepted cash. With co-pay, deductible and premium I could not afford that.* [Female, age 45 years] *I couldn’t afford the co pay and just quit taking the DMM [disease modifying medication]. I also quit taking other medications I could not afford to purchase.* [Female, age 64 years]
DMT access problems and related stress leads to multiple sclerosis relapses	*I’ve had a series of bad attacks when the prescription lapsed and when the insurance lapsed. I have some severe damage where it comes to process from the printed page and to spit it back out again. That ability is gone unless it’s in context…I had lesions confirmed. The area with the vocabulary. I’m also a bit slow on the processing. There are things I don’t do so well. It takes me that little extra moment, so there’s this pause in my conversation…Those are the two areas of the brain affected.* [Female, age 58 years] *You spend 45 min fighting on the phone it’s like working 8 hours. I have to take 2-3 hours of the day for a nap to get my energy back…I’ve done nothing but fight with [my insurance company].* [Female, age 51 years] *I had a relapse while waiting to get on [DMT]. My left arm is numb and tingling constantly.* [Female, age 54 years]
DMT access issues affect the quality of life	*When I didn’t have the medication, I have depression, and that’s not a surprise when you have MS, and it affected me really bad, especially without the [DMT] and I can’t afford it. I kept thinking I don’t know what’s happening inside of me and are things happening to me that I won’t be able to come back from? Not having the medication really affected me emotionally as well.”* [Female, age 59 years, on Medicare with too high co-pay] *My husband has to work 10 hour days and he’s stressed because of me. I worry about him. If I couldn’t get coverage on HC.gov, I could have gotten on my husband’s plan but that would have been more money, more money out of his paycheck. I went for the lesser of two evils but it’s still US $352 per month. There’s gotta be an in-between. I look for miracles. Someone that doesn’t qualify for disability there has to be some safety net.* [Female, age 51 years]
Personal resources enable access to overcome DMT barrier	*I had a 2-month delay to get the authorization. The prescription took over 30 days. First, they sent it to a retail pharmacy and it was rejected. Then it was sent to the wrong specialty pharmacy, and they denied it. Finally, after a month, I got the prescription. I used to do billing for Medicare, so I was familiar with pharmacy denials. Someone else would be lost, they would have had to do without their medicine. The fact that I got the prior authorization, I knew I didn’t have to pay full-price. I knew it should be covered under my plan.* [Female, age 64 years]

#### Egocentric Social Network of Disease-Modifying Therapy Access

**Figure 1:**
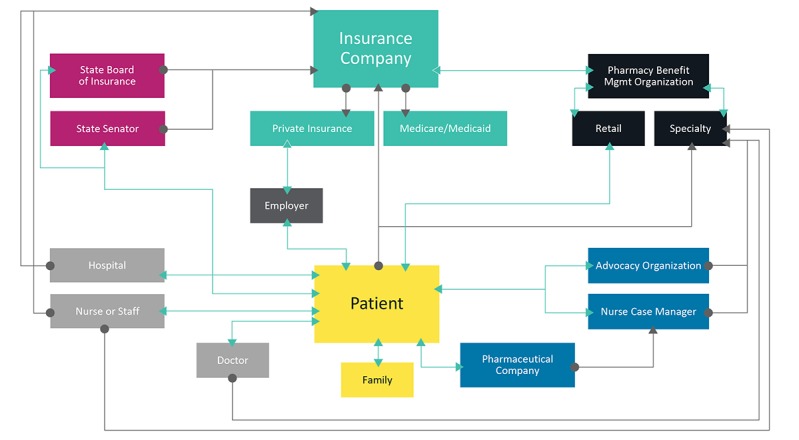
The aggregate egocentric social network of disease-modifying therapy access resolution based on 10 people with multiple sclerosis who experienced a relapse during their access issue.

Participants contacted numerous stakeholders during the process of obtaining a DMT. The complexity and intensity of work involved in resolving the problem placed a high burden on them. As one participant described, “I do all of the legwork” (Female, age 58 years). All interviewees contacted their insurance company, physicians, and specialty pharmacy while trying to obtain their DMT. Additional agents contacted included advocacy groups, pharmaceutical companies, government agencies, and hospitals. Figure 1 depicts a conceptual network visualization of these agents as a social network diagram.

Insurance-related access problems were attributed to changes in plans (eg, from an employer-sponsored plan to Medicare), formulary changes by insurance companies’ pharmacy benefit manager, or copayment payment policies. For example, a change to the pharmacy benefit manager contracted with one woman’s insurance company left her with different coverages, new step therapy requirements, and without access to her DMT for months at a time. In addition, insurance changes led to uncertainty about future access; for example, one participant could not find information about whether the Medicaid plan he would enter later in the year would cover his DMT. Finally, some participants could not afford to meet insurance requirements to pay the full price of DMT upfront and wait to be reimbursed later for the portion covered by the insurer.

Providers facilitated the documentation of proof of the medical need for advocacy groups or pharmaceutical company programs offering copayment assistance. For some, physician documentation was easily accomplished, while for others, it was a frustrating impediment that required additional calls or visits, and in some cases, delayed treatment access.

Barriers at pharmacies centered around finding a specialty pharmacy to work with their insurance and/or copayment assistance program. Resolving these issues could involve weeks of phone calls and research into plan coverages at specific pharmacies. Several participants reported that insurance companies were unable to provide accurate information about alternative pharmacies that might cover their DMT, forcing them to independently seek this information on the internet or elsewhere.

Nearly all interview participants had sought financial assistance from an advocacy group or a pharmaceutical company; this process was marked by complexity and logistical challenges requiring substantial investment of time and effort to prove need and coordinate stakeholders. Participants reported a range of experiences with patient advocacy groups, from helpful to challenging. Some reported no problems with obtaining copayment assistance from advocacy groups but experienced problems elsewhere in the access process. Most described the advocacy group funding assistance as a “grant” distributed for a certain total amount of money, after which point they would need to reapply. However, these organizations sometimes lacked sufficient funds to (re-)distribute. Others described spending dozens of hours on calls and paperwork to prove eligibility, with one woman reporting her income was deemed US $100 too high for assistance, despite living in an expensive area with a relatively modest income.

Pharmaceutical companies provided participants with copayment assistance or direct access to a DMT. Some participants had difficulty navigating the administrative paperwork necessary to access their copayment assistance programs, which could require original signatures that some found difficult to obtain.

Others reported that pharmaceutical companies’ copayment assistance programs were helpful facilitators, coordinating copayment assistance among patients, providers, and pharmacies or in one instance directly sending medication to a patient when the insurance company and the pharmacy failed to work with the pharmaceutical company’s copayment assistance program.

Two interviewees contacted government agencies to enforce the insurance coverage of DMTs that were not being followed; this required additional effort and knowledge to access services to enforce coverage of DMTs.

Finally, participants reported that their families provided emotional and financial support, working to provide insurance and encouraging them to keep trying until they obtained their DMT. However, several reported they felt they were a burden to their spouse or other family members.

## Discussion

### Principal Findings

In the United States, people with MS can face difficulty accessing DMTs because of insurance, pharmacy, or provider policies. This study demonstrated that issues related to DMT access occur frequently, commonly because of the need for authorizing the documentation, high out-of-pocket costs, and agency or provider coordination problems. Furthermore, participants reported that the effort to overcome barriers could be exceptionally time consuming, complex, and stressful for people with RRMS. This effort required contacting multiple stakeholders in their care administration, including providers, insurers, patient advocacy groups, and others. Furthermore, owing to the lack of DMT, people may navigate this complex process while experiencing disease progression and worsening symptoms. Some participants reported experiencing negative health outcomes during this lack of access, including relapse.

This work represents one of the few studies to both detail the frequency of DMT access issues as well as highlights the patient perspective throughout the DMT acquisition process and the impact this may have on their health outcomes. Furthermore, this study uses a novel approach, blending quantitative and qualitative methods to illuminate the patient experience with DMT access barriers from their own perspective. This approach offers depth and real-world insight that cannot be observed from administrative sources such as claims databases.

### Comparison With Prior Work

These findings confirm previous research showing that insurance-related access barriers can be associated with adverse outcomes, such as suboptimal adherence, which is associated with higher medical costs [35-41]. Similar to other studies with people with MS, participants in this study reported that before the DMT issue, many had to reduce or stop working because of functional cognitive decline related to MS [4-6,42,43]. Noting the burden of cost, paperwork, and benefit changes on people with MS trying to obtain DMT, medication advocacy organizations and provider groups have called for system-wide transparency, lower drug prices for DMTs, and policy reforms to assist people with MS with the cost burden of their care [8,44,45].

While the Affordable Care Act of 2010 was implemented to benefit patients by improving the overall health insurance access [46], many patients still lack access to DMTs because of their high cost and specialty status [14]. Cost-sharing efforts on the part of payers have forced many patients to seek financial assistance to defray the costs of DMTs [19,47]. This study showed that even when these programs are available, the logistics of taking part are complex, burdensome, and sometimes unsuccessful, leading to elevated stress levels and, potentially, relapse.

### Limitations

There are several limitations of our study which deserve mention. The generalizability of PatientsLikeMe patient population may not reflect the general population of people with MS as users of health-based internet sites are more likely to be female, younger, and more educated than those sampled from a clinic [48]. Results from interviews are not representative of all people with MS on PatientsLikeMe, nor those who completed the survey, as they were selected to include only those who experienced difficulty obtaining a DMT. Patient-reported explanations for DMT access difficulties are subject to errors in recall and errors in the reconstruction of events, especially among participants who reported MS relapse during the access issue. Finally, as the interview sample size was likely not sufficient to achieve concept saturation, resultant themes should be interpreted with caution.

### Conclusions

This study stresses the need for future research to incorporate the patient perspective to better understand barriers to MS treatment access. The evaluation of the long-term impact of DMT access barriers on patient and disease outcomes are needed. Formulary decision makers must consider the patient experience when making DMT coverage decisions. Clinicians should be aware of how patients experience DMT access difficulties and help deliver solutions to them when feasible. The MS patient experience with DMT access will continue to evolve with ongoing policy and payer landscape changes. Hence, frequent feedback from people with MS and stakeholders will be of paramount importance to ensure access to DMTs and to measure the associated impact on outcomes.

## Appendix

Multimedia Appendix 1 Survey questions.

Multimedia Appendix 2 Interview guide.

## Abbreviations

DMT: disease-modifying therapy
MRI: magnetic resonance imaging
MS: multiple sclerosis
PDDS: Patient-Determined Disease Step
RRMS: relapsing-remitting multiple sclerosis
