# Supplementary material for: The Impact of Disease-Modifying Therapy Access Barriers on People With Multiple Sclerosis: Mixed-Methods Study
Source: J Med Internet Res. 2018 Oct 30;20(10):e11168. doi: 10.2196/11168 (PMC6234348; doi:10.2196/11168)
Supplement: Multimedia Appendix 1 [file jmir_v20i10e11168_app1.pdf]

Study of Patient Access Experiences in MS

Let's get started with some basic questions:

Screener questions:

1.) do you live in the U.S.?
   a. Yes
   b. No

if no, end survey

2.) Have you been diagnosed with relapsing-remitting multiple sclerosis (MS) by a physician?
   a. Yes
   b. No

if no, end survey

3.) Were you ever prescribed a disease-modifying therapy (DMT)* for MS?
   a. Yes
   b. No
   c. I don't know

**DMTs are drugs such as Copaxone, Glatopa, Avonex, Rebif, Betaseron, Extavia, Plegridy, Tecfidera, Gilenya, Aubagio, Lemtrada, Novantrone, Tysabri that have been shown to alter the course of MS over a long period of time. DMTs don't include therapies that only help to control MS relapses, such as corticosteroids, adrenocorticotropic hormone, plasma exchange, or intravenous immunoglobulin. DMTs also don't include drugs that help control the symptoms of MS, such as Ampyra to aid with walking or stimulants that may aid with fatigue.*

if not yes, end survey

All questions

****

4.) When were you diagnosed with MS?
   a.) (year date picker)
   b.) I don't know

5.) How would you **best** describe your current work status? Please select one. (mult choice)
   a. Full time employment
   b. Part-time employment
   c. Retired
   d. Student
   e. Homemaker
   f. Unemployed
   g. Other

If not a or b, jump to Q7

6.) Using a 0-10 scale, where 0 is the worst job performance anyone could have at your job and 10 is the best, how would you rate your *usual* job performance?

Your usual job performance does not necessarily pertain to how you are performing your job right now or at any specific time, but in general. (numeric scale 0-10)

7.) Do you currently have a caregiver that helps you with daily activities? This person can be your spouse, child or another individual. (mult choice)

This person can be your spouse, child or another individual. (mult choice)

a. Yes
b. No

8.) Are you currently treating your MS with a disease-modifying therapy (DMT)*? (mult choice)

a. Yes
b. No
c. I don’t know

**DMTs are drugs such as Copaxone, Glatopa, Avonex, Rebif, Betaseron, Extavia, Plegridy, Tecfidera, Gilenya, Aubagio, Lemtrada, Novantrone, Tysabri that have been shown to alter the course of MS over a long period of time. DMTs don’t include therapies that only help to control MS relapses, such as corticosteroids, adrenocorticotropic hormone, plasma exchange, or intravenous immunoglobulin. DMTs also don’t include drugs that help control the symptoms of MS, such as Ampyra to aid with walking or stimulants that may aid with fatigue.*

If not yes, jump to Q12

9.) Which DMT are you currently taking to treat your MS? (mult choice picklist)

a. Copaxone (glatiramer acetate)
b. Glatopa (glatiramer acetate)
c. Avonex (interferon b-1a)
d. Rebif (interferon b-1a)
e. Betaseron (interferon b-1b)
f. Extavia (interferon b-1b)
g. Plegridy (pegylated interferon b-1a)
h. Tecfidera (dimethyl fumarate)
i. Gilenya (fingolimod)
j. Aubagio (teriflunomide)
k. Lemtrada (alemtuzumab)
l. Novantrone (mitoxantrone)
m. Tysabri (natalizumab)
n. Other: please specify_________

10.) How often do you typically take your DMT medication as prescribed? Given your DMT dosing schedule, please give your best estimate. (mult choice)

a. 0% of the time (never)
b. 10% of the time
c. 20% of the time
d. 30% of the time
e. 40% of the time

f. 50% of the time (about half the time)
g. 60% of the time
h. 70% of the time
i. 80% of the time
j. 90% of the time
k. 100% of the time (always)

If i,j,k, jump to Q12

11.) Please indicate why you sometimes do not take your DMT medication as prescribed. *Check all that apply*.

a. I forget to
b. To avoid side effects of the medication
c. I am feeling well so I don't think I need to
d. It's inconvenient
e. I don't feel like it or am tired of taking my DMT
f. I have injection anxiety
g. None of the above (exclusive)
h. Other, specify:____.

12.) How often do you practice each of the following self-care techniques? Self-care techniques are habits or behaviors that help alleviate the symptoms of MS or other conditions. This includes behaviors such as eating right, getting enough sleep, properly taking your medications, or exercising. (multi-item matrix, with likert response options: Never, Rarely, Sometimes, Often, all the time)

a. Eating right
b. Exercising
c. Getting enough sleep
d. Properly taking your medications
e. Taking vitamins/supplements
f. Meditation and other stress-relieving activities
g. Other ***if other is never jump to Q13, else…***
   Please specify the other self-care techniques you use to help alleviate the symptoms of MS or other conditions. ______________________________.

13.) Considering all parts of my life – physical, emotional, social, spiritual, and financial – over the past month, the quality of my life has been: (numeric scale with anchors: 0=very bad to 10=excellent)

14.) Please read the choices listed below and choose the one that best describes your own situation. **This scale focuses mainly on how well you walk**. You might not find a description that reflects your condition exactly, but please mark the **one** category that describes your situation the closest.

a. ☐ 0 Normal: I may have some mild symptoms, mostly sensory due to MS but they do not limit my activity. If I do have an attack, I return to normal when the attack has passed.

b. ☐ 1 Mild Disability: I have some noticeable symptoms from my MS but they are minor and have only a small effect on my lifestyle.
c. ☐ 2 Moderate Disability**:** I don't have any limitations in my walking ability. However, I do have significant problems due to MS that limit daily activities in other ways.
d. ☐ 3 Gait Disability**:** MS does interfere with my activities, especially my walking. I can work a full day, but athletic or physically demanding activities are more difficult than they used to be. I usually don't need a cane or other assistance to walk, but I might need some assistance during an attack.
e. ☐ 4 Early Cane: I use a cane or a single crutch or some other form of support (such as touching a wall or leaning on someone's arm) for walking all the time or part of the time, especially when walking outside. I think I can walk 25 feet in 20 seconds without a cane or crutch. I always need some assistance (cane or crutch) if I want to walk as far as 3 blocks.
f. ☐ 5 Late Cane**:** To be able to walk 25 feet, I have to have a cane, crutch or someone to hold onto. I can get around the house or other buildings by holding onto furniture or touching the walls for support. I may use a scooter or wheelchair if I want to go greater distances.
g. ☐ 6 Bilateral Support**:** To be able to walk as far as 25 feet I must have 2 canes or crutches or a walker. I may use a scooter or wheelchair for longer distances.
h. ☐ 7 Wheelchair / Scooter: My main form of mobility is a wheelchair. I may be able to stand and/or take one or two steps, but I can't walk 25 feet, even with crutches or a walker.
i. ☐ 8 Bedridden: Unable to sit in a wheelchair for more than one hour.

Text:
The next few questions are about DMT medication access issues- or times when you've had difficulties receiving or obtaining a DMT medication for MS. Please focus on times when you could not obtain a DMT medication as a result of policies or decisions made by your insurance company. These should not include times when you forgot to refill or take your DMT. Please focus only on DMT medications you've needed for multiple sclerosis.
Note: DMTs are drugs such as Copaxone, Glatopa, Avonex, Rebif, Betaseron, Extavia, Plegridy, Tecfidera, Gilenya, Aubagio, Lemtrada, Novantrone, Tysabri that have been shown to alter the course of MS over a long period of time. DMTs don't include therapies that only help to control MS relapses, such as corticosteroids, adrenocorticotropic hormone, plasma exchange, or intravenous immunoglobulin. DMTs also don't include drugs that help control the symptoms of MS, such as Ampyra to aid with walking or stimulants that may aid with fatigue.

15.) **Have you ever had** the following difficulties accessing or receiving DMT medication for MS? *Check all that apply*
a.) The medication was not covered by my insurance plan
b.) The medication was covered by my insurance plan, but it required authorizing documentation that I had to fill out or that my physician had to submit to my insurance
c.) The medication was covered by my insurance plan, but the out-of-pocket costs were too high

d.) The medication was covered by my insurance plan, but I was required to take at least one additional DMT medication before I could receive the one I was prescribed to take
e.) I did not have insurance
f.) I could not obtain it at my desired pharmacy or infusion center
g.) None of the above (exclusive)
h.) Other ____.

Branch
****
16.) Which option best describes your experience with accessing or receiving DMT treatment for MS?
   a. I am currently experiencing difficulty accessing DMT medication(s) ***if a, turn off modules "Past, Never"***
   b. I have had difficulties accessing DMT medication(s) in the past, but not currently ***if b, turn off modules "Current, Never"***
   c. I have never experienced difficulties accessing DMT medication(s) ***if c, turn off modules "Frequency, Past, Current, Interview"***

Frequency
****
17.) Since you received your MS diagnosis, about how many separate occasions have you had difficulty accessing any DMT medication for your MS?
   a. Numeric variable (range 1-n)
   b. I don't know
****
Past Questions
Text: The next set of questions is about the <u>most recent</u> time you had trouble accessing your MS DMT medication. While you may have had difficulties getting DMT medication before this, when asked about your medication access issue, focus only on your most recent experience accessing MS DMT medication.

18.) When was your **most recent** issue with accessing DMT medication? (month date picker)

19.) Thinking about your **most recent** DMT medication access issue, which medication did it concern? (mult choice)
   a. Copaxone (glatiramer acetate)
   b. Glatopa (glatiramer acetate)
   c. Avonex (interferon b-1a)
   d. Rebif (interferon b-1a)
   e. Betaseron (interferon b-1b)
   f. Extavia (interferon b-1b)
   g. Plegridy (pegylated interferon b-1a)
   h. Tecfidera (dimethyl fumarate)

i. Gilenya (fingolimod)
j. Aubagio (teriflunomide)
k. Lemtrada (alemtuzumab)
l. Novantrone (mitoxantrone)
m. Tysabri (natalizumab)
n. Other, please specify: ____________

20.) Thinking about your **most recent** medication access issue, did you receive a prescription for this DMT? (mult choice)

a. Yes
b. No

If b, jump to Q29

21.) Thinking about your **most recent** DMT access issue, what difficulties did you have accessing or receiving your DMT medication? *Check all that apply.*

a. The medication was not covered by my insurance plan
b. The medication was covered by my insurance plan, but it required authorizing documentation that I had to fill out or that my physician had to submit to my insurance
c. The medication was covered by my insurance plan, but the out-of-pocket costs were too high
d. The medication was covered by my insurance plan, but I was required to take at least one additional DMT medication before I could receive the one I was prescribed to take
e. I did not have insurance
f. I could not obtain it at my desired pharmacy or infusion center
g. I don't know why I did not receive or experienced a delay in receiving the prescribed DMT medication (exclusive)
h. Other ____.

If g, jump to Q23, else if not d jump to Q22

i. You mentioned that you were required to take at least one additional DMT medication before you could receive the one you were prescribed to take. Which option best describes your most recent situation? (check all that apply, randomized order)
    1. I was required to try other oral medication(s) before taking the prescribed oral DMT medication
    2. I was required to try injectable medication(s) before taking the prescribed oral DMT medication
    3. I was required to try infusion(s) before taking the prescribed oral DMT medication
    4. I was required to try other injectable medication(s) before taking the prescribed injectable DMT medication

5. I was required to try other oral medication(s) before taking the prescribed injectable DMT medication
6. I was required to try infusion(s) before taking my prescribed injectable DMT medication
7. I was required to try other oral medication(s) before taking the prescribed infusion DMT medication
8. I was required to try other injectable medication(s) before taking the prescribed infusion DMT medication
9. I was required to try other infusion medication(s) before taking the prescribed infusion DMT medication
10. Other ___________________.

Injectable	Oral	Infusion
Treatment that is given as a self-injectable (intramuscular or subcutaneous)<br>• Copaxone (glatiramer acetate)<br>• Glatopa (glatiramer acetate)<br>• Avonex (interferon b-1a)<br>• Rebif (interferon b-1a)<br>• Betaseron (interferon b-1b)<br>• Extavia (interferon b-1b)<br>• Plegridy (pegylated interferon b-1a)	Treatment that is given as a pill<br>• Tecfidera (dimethyl fumarate)<br>• Gilenya (fingolimod)<br>• Aubagio (teriflunomide)	Treatment that is given as an infusion through the vein<br>• Lemtrada (alemtuzumab)<br>• Novantrone (mitoxantrone)<br>• Tysabri (natalizumab)

22.) Thinking about your most recent DMT access issue, what was **the main reason** you had difficulty accessing or receiving your prescribed MS DMT medication?

a. The medication was not covered by my insurance plan
b. The medication was covered by my insurance plan, but it required authorizing documentation that I had to fill out or that my physician had to submit to my insurance
c. The medication was covered by my insurance plan, but the out-of-pocket costs were too high
d. The medication was covered by my insurance plan, but I was required to take at least one additional DMT medication before I could receive the one I was prescribed to take
e. I did not have insurance
f. I could not obtain it at my desired pharmacy or infusion center
g. Other ____.

if e or f, and Q21 includes none of a-d, jump to Q29

23.) Please select the option that best describes your situation at the **most recent** time you had difficulties obtaining your prescribed DMT. (mult choice)

a. This was my first experience with MS DMT medication
b. I had taken an MS DMT previously, but I was not taking a DMT at this time
c. I was taking a DMT for MS
d. I was taking a non-DMT medication for MS

If not c or d, jump to Q24

i. During your **most recent** medication access issue, how often did you take your MS medication as prescribed? (mult choice)
   1. 0% of the time (never)
   2. 10% of the time
   3. 20% of the time
   4. 30% of the time
   5. 40% of the time
   6. 50% of the time (about half the time)
   7. 60% of the time
   8. 70% of the time
   9. 80% of the time
   10. 90% of the time
   11. 100% of the time (always)

if 9-11, jump to Q24

ii. Please indicate why you sometimes did not take your MS medication as prescribed. *Check all that apply.*
   1. I forgot to
   2. To avoid side effects of the medication
   3. I was feeling well so I didn't think I needed to
   4. It was inconvenient
   5. I didn't feel like it or was tired of taking it
   6. I had injection anxiety
   7. None of the above
   8. Other, please specify:____.

24.) Pick the option that best describes how your **most recent** DMT medication access issue was resolved (mult choice):

a. I received my prescribed medication within a reasonable amount of time
b. I received another DMT medication before receiving my originally prescribed DMT medication
c. I went without medication until I received my prescribed DMT medication
d. I did not receive my originally prescribed medication, I was prescribed another DMT medication instead
e. I did not receive my prescribed medication, I continued taking my old medication
f. I did not receive my prescribed medication and did not treat with (any) medication
g. Other, please describe: _______________________________________

if not c-d jump to Q27, else if c jump to Q26

25.) You indicated that you received another medication which was not your **originally** prescribed DMT medication. Did you take or try this other medication?

a. Yes
b. No

if Q24 d, jump to Q27

26.) You indicated that you received your prescribed medication after some time without it. Approximately how long did you have to wait to receive it? (Numeric, unit in weeks)

27.) During the time you couldn't access your prescribed DMT medication, did you experience any MS relapses?

a. Yes
b. No

If not yes and Q24 is a-c jump to Q28, else if not yes and Q24 is not a-c jump to Q29

i. How many MS relapses did you have during the time you couldn't access your prescribed DMT medication?
   1. Numeric range- (1-n)

28.) (if 24 a, b, c) You indicated that you ultimately received your prescribed medication. Who was responsible for resolving the situation? *Check all that apply.*

a. Myself
b. My caregiver
c. Doctor, nurses, or office staff
d. Manufacturer (drug company)
e. Insurance company
f. Infusion center/clinic at a medical facility
g. Pharmacy/specialty pharmacy
h. Other, please specify ____.

29.) Please explain what happened during your **most recent** medication access issue and how the situation was resolved. (free text, include skip option)

____
____.

30.) Were you working for pay during your **most recent** DMT access issue? (Please include full-time or part-time employment which you were paid to do.)

a. Yes
b. No

If not yes jump to Q31

i. Using a 0-10 scale, where 0 is the worst job performance anyone could have at your job and 10 is the best, how would you rate your job performance during the time that you were having difficulty receiving DMT medication? (numeric scale 0-10)

31.) Did you have a caregiver during your medication access issue?

a. Yes
b. No

If not a jump to Q32

i. Did your caregiver help you to obtain your prescribed DMT medication? (This may have included help making phone calls, going to the pharmacy, or filling out forms.)
   1. Yes
   2. No

If not 1, jump to Q32

ii. To what extent do you think your medication access issue affected your caregiver's workload compared to other times when you did not have an MS medication access issue?
   1. No effect
   2. Minor effect
   3. Neutral
   4. Moderate effect
   5. Major effect

32.) On a scale from 0-10, where 0 is not at all and 10 is greatly, how stressful has your most recent DMT medication access issue was? (numeric, 0-10)

33.) Considering all parts of my life – physical, emotional, social, spiritual, and financial – during the time of my medication access issue, the quality of my life was: Use a 0-10 scale, where 0 is the worst it has ever been and 10 is the best. (numeric scale with 2 anchors: 0=very bad to 10=excellent)

34.) During your medication access issue, how often did you take your other, **non-MS prescription medication(s)** as prescribed? Given your medication dosing schedule, please give your best estimate. (mult choice)

c. 0% of the time (never)
d. 10% of the time
e. 20% of the time
f. 30% of the time

g. 40% of the time
h. 50% of the time (about half the time)
i. 60% of the time
j. 70% of the time
k. 80% of the time
l. 90% of the time
m. 100% of the time (always)
n. Does not apply

35.) **During your medication access issue**, how often did you practice each of the following self-care techniques? Self-care techniques are habits or behaviors that help alleviate the symptoms of MS or other conditions. It includes things like eating right, getting enough sleep, properly taking your medications, and exercising. (multi-item matrix, with likert response options: Never, Rarely, Sometimes, Often, all the time)

o. Eating right
p. Exercising
q. Getting enough sleep
r. Properly taking your medications
s. Taking vitamins/supplements
t. Meditation and other stress-relieving activities
u. Other ***if other is never jump to Q37, else…***
Please specify the other self-care techniques you use to help alleviate the symptoms of MS or other conditions. ______________________________.

36.) What advice do you have for other MS patients who are having issues receiving or obtaining their DMT medication(s)? Can you suggest any strategies that worked for you? (free text and I prefer to skip)
____.

Current Questions

*Text: The next set of questions is about your **current** trouble accessing your MS DMT medication. While you may have had difficulties getting DMT medication before this, when asked about your medication access issue, focus only on your most recent experience accessing MS DMT medication.*

17.) When did you first start experiencing difficulty accessing or receiving your DMT medication?

a. Numeric date mm/dd/yyyy

18.) Which DMT medication are you having difficulty accessing? (mult choice)

a. Copaxone (glatiramer acetate)
b. Glatopa (glatiramer acetate)
c. Avonex (interferon b-1a)
d. Rebif (interferon b-1a)
e. Betaseron (interferon b-1b)
f. Extavia (interferon b-1b)

g. Plegridy (pegylated interferon b-1a)
h. Tecfidera (dimethyl fumarate)
i. Gilenya (fingolimod)
j. Aubagio (teriflunomide)
k. Lemtrada (alemtuzumab)
l. Novantrone (mitoxantrone)
m. Tysabri (natalizumab)
n. Other, please specify: ___________

19.) Did you receive a prescription for this DMT? (mult choice)

a. Yes, I received a prescription for this DMT
b. No, I did not receive a prescription for this DMT

If not a, jump to Q25

20.) What difficulties **are you having** accessing or receiving your DMT medication? (check all that apply)

a. The medication is not covered by my insurance plan
b. The medication is covered by my insurance plan, but it requires authorizing documentation that I have to fill out or that my physician has to submit to my insurance
c. The medication is covered by my insurance plan, but the out-of-pocket costs are too high
d. The medication is covered by my insurance plan, but I am required to take at least one additional DMT medication before I can receive the one I was prescribed to take
e. I do not have insurance
f. I could not obtain it at my desired pharmacy or infusion center
g. I don't know why I cannot receive or am experiencing a delay in receiving the prescribed DMT medication (exclusive)
h. Other, please specify ____.

If response g jump to Q22, else if response not d jump to Q21

i. You mentioned that you are required to take at least one additional DMT medication before you can receive the one you were prescribed to take. Which option best describes your most recent situation? (check all that apply, randomized order)
    11. I am required to try other oral medication(s) before taking the prescribed oral DMT medication
    12. I am required to try injectable medication(s) before taking the prescribed oral DMT medication

13. I am required to try infusion(s) before taking the prescribed oral DMT medication
14. I am required to try other injectable medication(s) before taking the prescribed injectable DMT medication
15. I am required to try other oral medication(s) before taking the prescribed injectable DMT medication
16. I am required to try infusion(s) before taking my prescribed injectable DMT medication
17. I am required to try other oral medication(s) before taking the prescribed infusion DMT medication
18. I am required to try other injectable medication(s) before taking the prescribed infusion DMT medication
19. I am required to try other infusion medication(s) before taking the prescribed infusion DMT medication
20. Other, please specify___________________.

Injectable	Oral	Infusion
Treatment that is given as a self-injectable (intramuscular or subcutaneous)<br>• Copaxone (glatiramer acetate)<br>• Glatopa (glatiramer acetate)<br>• Avonex (interferon b-1a)<br>• Rebif (interferon b-1a)<br>• Betaseron (interferon b-1b)<br>• Extavia (interferon b-1b)<br>• Plegridy (pegylated interferon b-1a)	Treatment that is given as a pill<br>• Tecfidera (dimethyl fumarate)<br>• Gilenya (fingolimod)<br>• Aubagio (teriflunomide)	Treatment that is given as an infusion through the vein<br>• Lemtrada (alemtuzumab)<br>• Novantrone (mitoxantrone)<br>• Tysabri (natalizumab)

21.) What is **the main reason** you are having difficulty accessing or receiving your prescribed MS DMT medication? (mult choice)

a. The medication is not covered by my insurance plan
b. The medication is covered by my insurance plan, but it requires authorizing documentation that I have to fill out or that my physician has to submit to my insurance
c. The medication is covered by my insurance plan, but the out-of-pocket costs are too high
d. The medication is covered by my insurance plan, but I am required to take at least one additional DMT medication before I can receive the one I am prescribed to take
e. I do not have insurance
f. I could not obtain it at my desired pharmacy or infusion center

g. I don’t know why I cannot receive or experienced a delay in receiving the prescribed DMT medication
h. Other, please describe ____.

If e or f and Q20 includes none of a-d… skip to Q31

22.) What other MS medication(s) are you taking while your DMT medication access issue is being resolved? (mult choice)

a. I am continuing to take my old non-DMT medication
b. I am continuing to take my old DMT medication
c. I am taking a newly prescribed other DMT medication
d. I am taking a newly prescribed other non-DMT medication
e. I am not taking any other MS medications

If not a-d jump to Q24

23.) You indicated that you are currently taking a medication other than your prescribed DMT medication. During your medication access issue, how often do you take your MS medication as prescribed?

a. 0% of the time (never)
b. 10% of the time
c. 20% of the time
d. 30% of the time
e. 40% of the time
f. 50% of the time (about half the time)
g. 60% of the time
h. 70% of the time
i. 80% of the time
j. 90% of the time
k. 100% of the time (always)

if i-k, jump to Q24

i. Please indicate why you sometimes do not take your MS medication as prescribed. *Check all that apply.*

1. I forget to
2. To avoid side effects of the medication
3. I am feeling well so I don’t think I needed to
4. It is inconvenient
5. I don’t feel like it or I am tired of taking it
6. I have injection anxiety
7. None of the above
8. Another reason, please describe:____.

24.) Have you experienced any MS relapse(s) since you began having difficulty accessing your prescribed DMT medication?

a. Yes
b. No

If not a jump to Q25

i. How many MS relapses have you had?
   1. Numeric range- (1-n)

25.) How do you hope to resolve your current DMT access issue? (free text) ____.

If Q5 is not a or b and Q7 is not a, jump to Q28 else if Q5 is not a or b and Q7 is a jump to Q27

26.) You indicated that you are currently working for pay. On a 0-10 scale, where 0 is the worst job performance anyone could have at your job and 10 is the best, how would you rate your current job performance at the current time? (numeric scale 0-10)

27.) You indicated that you currently have a caregiver. Is your caregiver helping you to obtain your desired DMT medication? (This can include making phone calls, going to the pharmacy, or helping fill out forms).

a. Yes
b. No

If not yes, jump to Q28

i. To what extent do you think your current medication access issue has affected your caregiver's workload?
   1. No effect
   2. Minor effect
   3. Neutral
   4. Moderate effect
   5. Major effect

28.) On a scale from 0-10, where 0 is not at all and 10 is greatly, how stressful has your current DMT medication access issue been? (numeric 0-10)

29.) Since the start of your access issue, how often would you say you are taking your other, **non-MS prescription medication(s)** as prescribed? Given your medication dosing schedule, please give your best estimate (mult choice)

a. 0% of the time (never)
b. 10% of the time

c. 20% of the time
d. 30% of the time
e. 40% of the time
f. 50% of the time (about half the time)
g. 60% of the time
h. 70% of the time
i. 80% of the time
j. 90% of the time
k. 100% of the time (always)
l. Does not apply

30.) **Since the start of your access issue**, how often would you say you have been able to practice self-care compared to your normal self-care routine? Self-care techniques are habits or behaviors that help alleviate the symptoms of MS or other conditions. It includes things like eating right, getting enough sleep, properly taking your medications, and exercising. (multi-item matrix, with likert response options: Never, Rarely, Sometimes, Often, all the time)

a. Eating right
b. Exercising
c. Getting enough sleep
d. Properly taking your medications
e. Taking vitamins/supplements
f. Meditation and other stress relieving activities
g. Other ***if never, jump to Q31***
Please specify the other self-care techniques you use to help alleviate the symptoms of MS or other conditions. _____________________________.

31.) If you could ask other MS patients about their issues with accessing DMT medication, what would you say? What is it that you would you most like to know, and which types of issues would you most like to be addressed and resolved? (free text with I prefer to skip)

____.

Never Questions
****

17.) When did you experience your latest MS relapse?

a. (answer choices to include past year to 10 years or more)

18.) On a scale from 0-10, where 0 is not at all and 10 is greatly, how stressed have you been in the past 7 days?

19.) How often do you take your other, **non-MS prescription medication(s)** as prescribed? Your other prescription medications do not have to be for MS (mult choice)

a. 0% of the time (never)
b. 10% of the time
c. 20% of the time
d. 30% of the time
e. 40% of the time
f. 50% of the time (about half the time)
g. 60% of the time
h. 70% of the time
i. 80% of the time
j. 90% of the time
k. 100% of the time (always)

Anchor Questions

****

37.) How confident are you filling out medical forms by yourself? (5 pt likert mult choice).

a. Not at all
b. A little
c. Somewhat
d. Quite a bit
e. Extremely

38.) What is your marital status? (no randomize)

v. Married/cohabitating
w. Divorced
x. Separated
y. Widowed
z. Single/Never married
aa. Other: ________________
bb. I prefer to skip

39.) What is your yearly household income?

1. Less than $20,000
2. $20,000 to $34,999
3. $35,000 to $49,999
4. $50,000 to $74,999
5. $75,000 to $99,999
6. $100,000 to $149,999
7. $150,000 to $199,999
8. $200,000 or more

9. I prefer to skip

Interview Questions

****

40.) Thank you for completing this survey. We really appreciate your time and are interested in hearing more about your thoughts and experiences during the time you had difficulties obtaining your DMT. **Would you be interested in a phone interview (up to 1 hour) with a PLM staff member to discuss your DMT access experiences?**

cc. Yes
dd. No

****

Matched platform variables: health insurance type, gender, age, race, educational level.
