# Supplementary material for: The Impact of Disease-Modifying Therapy Access Barriers on People With Multiple Sclerosis: Mixed-Methods Study
Source: J Med Internet Res. 2018 Oct 30;20(10):e11168. doi: 10.2196/11168 (PMC6234348; doi:10.2196/11168)
Supplement: Multimedia Appendix 2 [file jmir_v20i10e11168_app2.pdf]

MS Patient Access to DMTs – Interview Guide

Thanks so much for taking the time to talk to us today. Is this still a good time for you to talk to us?

I'm XX, I'm a member of the research team — it's always a pleasure to have a chance to speak to our members, so thanks for fitting us in.{I'm joined by a couple of colleagues at PatientsLikeMe }

Have you read and understood the consent form we sent you?

The goal of this interview is to understand more about problems you might have had filling a prescription for DMT treatment for your MS [this includes having to fill out paperwork to fill your prescription related to insurance authorization, having high out of pocket costs as a result of your prescription, your insurance not covering your prescription, or some other related difficulty]. It will last between 45 minutes and 1 hour. As we go through this interview, keep in mind you don't need to answer any questions that might make you uncomfortable, and please let us know if you if need a break or if you have any questions at all

Does this sound okay to you? Okay, then let's begin!

Intro & Background	***To start, could you tell us the 5 minute version of your MS story?***<br>o ***Probe - How did you find PatientsLikeMe?***<br>o ***Probe - When were you diagnosed with relapsing-remitting multiple sclerosis?***
Medication Access Journey: DMT medication experiences, occurrence of any access issues and	***On the survey, you talked a bit about the [current/most recent] time you had a problem or problems filling your DMT prescription for MS. We would like to walk through how the problem started, the steps that were taken along the way, and how it was eventually resolved.***<br><br>***What types of problems did you have when you tried to fill this prescription this most recent time?***<br><br>***IF PREAUTHORIZATION ISSUE:***

how they were resolved	*(Q15b: Probe)- [In the survey, you mentioned that you had to get prior authorization or documentation to receive the DMT you were prescribed. Could you tell me more about the type of documentation that was needed?* *Where did you have to go to get the paperwork?* *Who was responsible for getting the paperwork?* *Who filled out the paperwork?* *Where/who did you have to submit it to?* *What happened after the documentation was submitted?* *Did you have to send in any other information??* *How long did you have to wait for your DMT prescription throughout this process?* *IF COST ISSUE* *(Q15c: Probe)- You mentioned in the survey that the out of pocket costs for your DMT were too high. Did you eventually find a way to get the DMT?* *(if yes) Did you get help with the copay?* *(if yes) Which kind of copay program did you participate in?* *(if no to copay) how did you pay for it?)* *IF PRIOR TREATMENT ISSUE* *(Q15d: Probe):[You mentioned in the survey that you needed to take at least one additional DMT medication before you could take the one you were prescribed to take. How many additional DMTs did you need to take in total?* *What were they?* *How long were you on each?* *[add])(We will ask you about clinical consequences you may have experienced as a result of this issue later.)* *IF LACK OF INSURANCE ISSUE* *(Q15e: Probe): [You mentioned in the survey that you did not have insurance. Were you ever able to get the DMT which you were prescribed?]* *(If yes) how did you get it?*

	• *(if yes) How did you pay for it?*<br>• *(if no) What did you take instead?*
Agents	***Next I'm going to ask you about some different people or companies that might have been involved when you tried to fill your DMT prescription.***<br><br>***Did your insurance company give you any problems filling or paying for the prescription? What happened?***<br><br>***Did the pharmacy prevent you from filling the DMT prescription? What happened?***<br><br>***Did your doctor help you to sort out your DMT prescription problem?***<br>• ***What kind of help did they provide?***<br><br>***Did anyone else help you get your DMT treatment and medication? (can be more than one person)***<br>• ***Probe - in what specific way did they help you?***<br><br>***Did you end up receiving your medication?***<br>***(If yes) Walk me through the steps it took for you to receive your medication and how long the process took.***
Clinical Consequences	***You mentioned in the survey that you had at least one MS relapse while you were still dealing with getting your medication.***<br>• ***What happened as a result of this relapse?***<br>• ***Did a neurologist or other HCP confirm you had a relapse?***<br>• ***Did you go to the hospital as a result of this relapse?***<br>○ ***(if yes, for how long?)***<br>• ***Did you have an MRI scan done?***<br>○ ***(if yes, were any new lesions confirmed as a result of the***

	relapse?)<br>● ***Did you need to take either IV or oral steroids to control your relapse?)***<br>○ ***(If yes, for how long did you take steroids?)***<br>● ***After this relapse(s), did you experience any new symptoms (either of your MS or any other condition?)***<br>● ***(if prescribed another Rx): Did your doctor think the relapse was due to being off treatment/on a less effective treatment? Did you?***<br><br>***Did you ever have flare-ups or worsening of any of your other condition(s) when you were having difficulty getting your MS medication?***<br>● ***If yes, what happened?***
Impact on Patient (as a result of issue)	***Thinking about the process of trying to get your DMT medication, I'd like to know how that problem impacted the rest of your life; like work, relationships, or your other health concerns. How would you say the prescription problem impacted your life?***<br><br>● ***Optional Probe:.....on your work life?***<br>● ***Optional Probe: …on social life/relationships?***<br>● ***Optional Probe: …on health?***<br>● ***Optional Probe: …on overall well-being (quality of life?)***<br><br>***(Also ask about:)***<br>● ***Costs***<br>o ***Impact on financial situation- costs you may have accrued in terms of transportation, costs of new medication, administrative costs, other costs [direct costs]***<br>o ***Resources used or time spent trying to resolve the issue (yours or other's time, energy) [indirect costs]***<br>o ***Do you have any health concerns other than relapsing-remitting multiple sclerosis?***<br>o ***(If yes) Do any of your other conditions also require medication that can be costly or puts a***

	strain on your budget?
Anything else	***Is there anything else you would like to add?***

Thanks so much for taking the time to talk to us. We'll be using what we learn here to help our partner better understand what MS patients are experiencing trouble filling DMT medication prescriptions. Thank you again and look out for a give-back on the survey results from the survey you took a few weeks ago.
